# Assessing the developmental effects of fentanyl and impacts on lipidomic profiling using neural stem cell models

**DOI:** 10.3389/ebm.2025.10607

**Published:** 2025-06-25

**Authors:** Cheng Wang, Jinchun Sun, Rohini Donakonda, Richard Beger, Leah E. Latham, Leihong Wu, Shuliang Liu, Joseph P. Hanig, Fang Liu

**Affiliations:** ^1^ Division of Neurotoxicology, National Center for Toxicological Research/FDA, Jefferson, AR, United States; ^2^ Division of Systems Biology, National Center for Toxicological Research/U.S. Food and Drug Administration (FDA), Jefferson, AR, United States; ^3^ Division of Bioinformatics and Biostatistics, National Center for Toxicological Research/FDA, Jefferson, AR, United States; ^4^ Office of Pharmaceutical Quality, Center for Drug Evaluation and Research/U.S. Food and Drug Administration (FDA), Silver Spring, MD, United States

**Keywords:** development, fentanyl, lipidomic analysis, anesthetics, neurotoxicity

## Abstract

Fentanyl is a potent and short-acting opioid that is often given to pediatric patients during surgery to relieve pain and as an adjunct to anesthesia. Its effects on the developing brain are yet to be determined. In the present study, commercially available human neural stem cells (NSCs) were used to model the effects of fentanyl on the developing human brain. We determined the dose dependent effects and temporal relationships between fentanyl exposures and NSC health, viability, and differentiation. Markers of mitochondrial health [3-(4,5-dimethylthiazol-2-yl)-2,5-diphenyltetra-zolium bromide (MTT)] and cell death/damage [lactate dehydrogenase (LDH)] were monitored to determine the dose response effects of fentanyl on NSC viability. In addition, lipidomics analysis was conducted to investigate lipid profile changes in differentiated neural cells treated with fentanyl. Fentanyl did not cause a significant increase in LDH release, nor MTT reduction after 24-h exposure at concentrations of 0.5, 1.0, 3.0, 10, or 100 μM, for both NSCs and differentiated neural cells. Lipidomics data showed the top 15 most variable important in projection (VIP) lipid species (the higher the VIP scores, the bigger changes in treated groups vs. controls), including lysophosphatidylcholines (LPCs), lysophosphatidylethanolamines (LPEs), ceramides (CER), cholesterol esters (ChEs) and sphingosine (SPH). The lipidomic data indicate that LPC (16:0), LPC (16:1), LPC (18:1), CER (d18:0_22:0), CER (d18:2_18:0), CER(d18:2_24:1) were significantly increased, and only ChE (24:5) and SPH (d18:1) were significantly decreased in the highest dose group versus control. These data indicated that fentanyl exposure (24-h) did not induce detectable cell death. However, a lipidomic analysis indicated that fentanyl may affect immature neural cell functions through modifying lipid composition and lipid metabolism. These data indicated that despite the absence of clear neurodegeneration, fentanyl may still have a negative impact on the developing brain.

## Impact statement

Opioids, such as fentanyl, are used as pediatric analgesics in pain management. Our data indicate/provide important information to understand how to continue to use these medications safely. In the present study, advanced lipidomic analysis using ultra-high-performance liquid chromatography coupled with high-resolution mass spectrometry was utilized to investigate underlying mechanisms and the impacts of different doses of fentanyl on NSCs and neural cells differentiated from NSCs. Although markers of neurotoxic assays showed that no detectable cell death occurred after fentanyl exposure at micromolar concentrations for 24 h, lipidomic analysis indicated that fentanyl may affect immature neural cell functions through modifying lipid composition and lipid metabolism. These data indicated that despite the absence of clear neurodegeneration, fentanyl may still have a negative impact on the developing brain, and changes in lipid composition may help explain the progression of neurodegeneration and could ultimately provide therapeutic/neuroprotective potential.

## Introduction

Opioids, including natural compounds, semi-synthetic and synthetic derivatives, are used as analgesics in pain management [1]. Children may be prescribed opioids to control severe pain resulting from their operations or injuries [2]. Opioids decrease severe pain by blocking pain signals in the brain and spinal cord [3]. Public concerns of prescription opioids include misuse, abuse, addiction, overdose, and death from respiratory depression [4, 5]. Also, fatal poisonings from opioid overdoses are increasing among children and teens [6]. It is important to understand how to continue to use these medications safely.

The most prescribed opioid analgesic (non-oral) in pediatric hospitalizations is fentanyl [7, 8]. Fentanyl is a lipid-soluble synthetic opioid and a strong mu receptor agonist with less respiratory suppression compared to morphine [9, 10]. It is a potent, short-acting opioid medication that is often given to pediatric patients during surgery to relieve pain and as an adjunct to anesthesia [6]. Despite the frequency of its use in young children, little is known about the effects of fentanyl on the developing brain. Due to the complexity and temporal dynamics of the developing nervous system, it is difficult to determine the adverse effects of fentanyl on human infant and children brain development [11–16]. However, application of highly relevant preclinical models, such as NSCs derived from humans, might serve as a bridging model to evaluate the sensitivity/vulnerability of the developing nervous system, and to address FDA’s regulatory needs.

Human NSCs can mimic or model particular developmental stages of the human brain, thus, providing a valuable model for conducting systematic dose-dependent response and time-course studies. Application of this approach may reduce the number of animals, and the amount of time/money required for developmental neurotoxicity assessments. In this study, commercialized hippocampus (HIP) NSCs from a fetal human brain were used to evaluate the vulnerability of the developing nervous system. The transformation of NSCs to neurons and glial cells occurs in two basic steps: 1) undifferentiated and proliferative NSCs replicate to form small clusters of cells; and 2) differentiation into neurons, astrocytes, and oligodendrocytes. Our previous data demonstrated that NSC viability and their ability to self-renew could be affected when NSCs were exposed to general anesthetics at relatively high concentrations [12], suggesting NSCs may be a good platform for evaluating developmental neurotoxicity. Markers of mitochondrial health (MTT Assay) and cell death/damage (LDH Release) were monitored to determine the dose-related effects of fentanyl on NSCs and differentiated cells.

General anesthetics (including a range of structurally diverse inhaled and injectable compounds) are highly lipid soluble and can dissolve in every membrane, penetrate organelles, and interact with numerous cellular constituents. Their actions have long been considered rapid and fully reversible, within the pharmacodynamic time course of anesthesia [17]. Although in most patients physiologic homeostasis is restored soon after general anesthesia, anesthetics have potentially profound and long-lasting effects that, in animal models, seem particularly consequential in specific developmental periods and pathophysiologic contexts [17]. Lipids are essential for cellular functioning considering their role in membrane composition, signaling, and energy metabolism. Neural cells contain a wide variety of lipid classes and lipid species in the brain. Abnormal lipid constitution and changes in their metabolic rates could be related to earlier stages of neural degenerative disorders and neural damage [11, 18–20]. In the central nervous system (CNS) lipid dysregulation has been linked to etiology, progression, and severity of neurodegenerative diseases/disorders including prolonged anesthetic-induced neurotoxicity [11–13]. Thus, changes in lipid composition may help explain the progression of neurodegeneration and could ultimately provide therapeutic/neuroprotective potential [11, 18, 21]. In the present study, lipidomic analysis using ultra-high-performance liquid chromatography (UHPLC) coupled with high-resolution mass spectrometry (HRMS) was utilized to investigate underlying mechanisms and the impacts of different doses of fentanyl on NSCs and neural cells differentiated from NSCs.

## Materials and methods

### Test chemicals

Fentanyl was purchased from Sigma Aldrich (St. Louis, MO), and freshly dissolved in culture media (VESTA Biotherapeutics) upon the experiments.

### Cultures

Commercially available, de-identified human NSCs derived from human fetal brains were utilized (VESTA Biotherapeutics). Media for NSC proliferation (named Neural StemCell Growth Medium) and for NSC differentiation (named Neural Differentiation Medium) were purchased from the same vendor (VESTA Biotherapeutics). The cells were seeded on laminin-coated dishes. Briefly, cultured cells on slides or in flasks were placed in a low oxygen (5% oxygen) culture incubator with humidified air and 5% carbon dioxide at 37°C. The culture media was changed every 3 days. The concentrations of oxygen and carbon dioxide in the chamber were continuously monitored. Control experiments were performed in the same manner. Cultured NSCs and/differentiated neural cells were exposed to fentanyl at 0.5, 1, 3, 10, and 100 µM [22] respectively, for 24 h.

### Assessment of neurotoxicity

#### MTT assay

The MTT dye is metabolized by viable mitochondria forming a colored product that can be dissolved in dimethyl sulfoxide (DMSO) and detected photometrically. Thus, the extent of MTT metabolism is an indicator of mitochondrial function. Briefly, after the removal of media for use in the lactate dehydrogenase (LDH) assay, 100 µL MTT (5 mg/10 mL of medium) was added to each well, and the plate was incubated for 2 h at 37°C. The MTT solution was removed and followed by the addition of 100 µL of DMSO to each well. The color intensity was assessed with a plate reader at 590 nm, as previously described [14, 23].

#### LDH release

LDH is a cytoplasmic enzyme retained by viable cells with intact plasma membranes. The release of LDH into the cell culture medium occurs with loss of plasma membrane integrity, a process most often associated with acute cell death [12]. After exposure (24 h) to different concentrations of fentanyl, the media were collected and assayed for LDH activity using a cytotoxicity detection kit from Roche Applied Science (Indianapolis, IN). Briefly, LDH catalyzes the conversion of lactate to pyruvate upon reduction of NAD^+^ to NADH/H^+^; the added tetrazolium salt (yellow) is then reduced to formazan (red). The amount of formazan formed correlates to LDH activity. The formazan product was measured with a plate reader at 490 nm, as previously described [14, 23].

### Lipidomic methods

#### Sample preparation (lipid extraction)

Protein content was used to normalize the determined lipid levels for the individual samples. LC/MS grade water (1 mL) was added to the Eppendorf tube containing differentiated cells (∼1 M counts), followed by vortexing for 40 s. The cell suspension was then transferred to glass tubes for lipid extraction. Lipid extraction was achieved using a modified version of the Bligh and Dyer extraction protocol [24], whereby 2 mL methanol and 0.9 mL dichloromethane (DCM) were added to the 1 mL cell suspension and mixed gently, but thoroughly for 5 s. Aliquots of stable internal standard mixtures-SPLASH^®^ Lipidomix^®^ Mass Spec Standard, which contained 14 individual isotope-labeled standards that cover 14 lipid classes, were spiked into all samples. Following two rounds of extraction, the bottom layers were combined and dried under nitrogen flow and reconstituted in 1 mL ethanol and centrifuged just prior to analysis.

#### Quality control in open-profiling lipidomics

Pooled lipid extracts were run every 10 sample injections by UHPLC/Exploris 240 MS to monitor the analytical equipment variability, also were used for data filtering as described in the raw data analysis.

#### UHPLC/HRMS analysis

Lipid extract (4 µL) was separated using a Thermo Accucore C30 column on a Thermo Vanquish Ultimate 3000 UPLC (Thermo Scientific, Waltham, MA). Chromatography was operated at a flow rate of 0.4 mL/min, and the column was maintained at 40°C during a 30 min gradient. The mobile phase consisted of solvent A (5 mM ammonium formate in 60% acetonitrile with 0.1% formic acid) and solvent B (5 mM ammonium formate in 10% acetonitrile and 90% isopropanol with 0.1% formic acid). Lipids were eluted using linear gradients of 40–55% solution B from 0 to 7 min, 55–65% solution B from 7 to 8 min, maintained at 65% B until 12 min, 65–95% solution B from 12 to 20 min, 95–100% solution B from 20 to 22 min and maintained at 100% B util 27 min, and finally returned to 40% B at 27.1 min.

Mass spectrometric (MS) data were collected with a Thermo Orbitrap Exploris 240 mass spectrometer (Thermo Scientific, Waltham, MA) operated in positive and negative ionization electrospray modes. Data were acquired in full-scan mode (*m/z* 70–1,000) at a resolution of 120,000 for all samples. The capillary voltage was set to 3.5 kV for positive ionization mode and 2.5 kV for negative ionization mode. Other parameters used for the data collection were an ion transfer tube temperature of 325°C, a vaporizer temperature of 350°C, sheath gas (arb) 50, auxiliary gas (arb) 10, and sweep gas (arb) 1. Internal mass calibration EASY-IC was used for mass accuracy. MS/MS data was collected in a sequence of separate runs operated by the intelligence-driven software AcquireX (Thermo Scientific) to acquire more MS/MS spectra from the detected ion features than data-dependent acquisition. The raw data were processed using LipidSearch (vers. 5.0; Thermo Scientific). Data were filtered using pooled QC samples based on the following criteria: *i)* ions with %RSD less than 30% in the pooled QC samples were included, *ii)* ions present in ≥70% of QC samples were included.

### Statistical analysis

Each condition/experiment, including lipidomics, was assessed in triplicate, and experiments were repeated three times independently. For the LDH and MTT assays, statistical analyses were performed, and graphs generated using SigmaPlot. Data were analyzed using one-way ANOVA followed by Dunnett’s *post hoc* test and expressed as mean ± SD. Significance was considered at a *p* value <0.05.

For lipidomics, the resulting dataset from LipidSearch processing was further analyzed by supervised partial least squares discriminant analysis (PLS-DA) using MetaboAnalyst v. 6.0^1^ [25]. The values in the treated group were compared to their respective control group, as mean ± SD. A value of *p* < 0.05 from the unpaired *t-*Test was considered statistically significant. Lipid intensity data was log-transformed prior to PLS-DA. Repeated measures ANOVA were performed using MetaboAnalyst v. 6.0^1^.

## Results

### Characterization of the *in vitro* models

**FIGURE 1:**
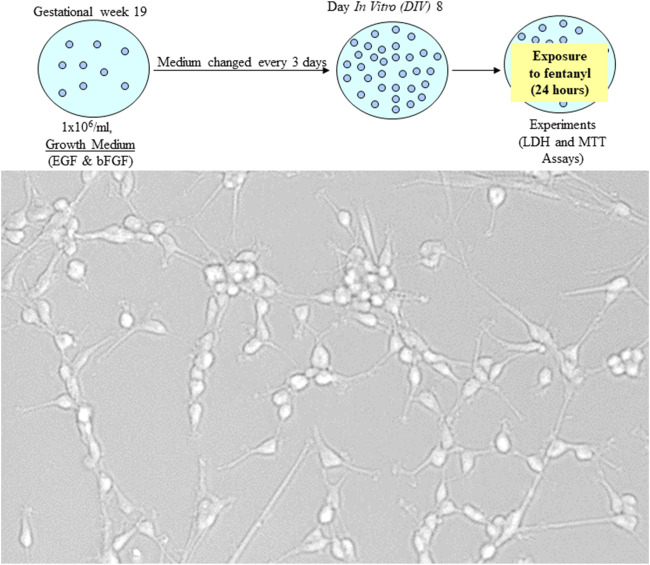
NSCs are multipotent cells in the nervous system. They have the features of being able to self-renew and give rise to differentiated progenitor cells to generate lineages of neurons as well as glia, such as astrocytes and oligodendrocytes. Human NSCs were seeded at a cell density of 1 × 10^6^/mL. When the cultures were maintained in the growth medium, the bipolar NSCs continuously proliferated.

The cultured NSCs were typically bipolar in shape. More and more cells were generated and gathered when the cultures were maintained in NSC growth media, demonstrating their capability of proliferation (Figure 1).

**FIGURE 2:**
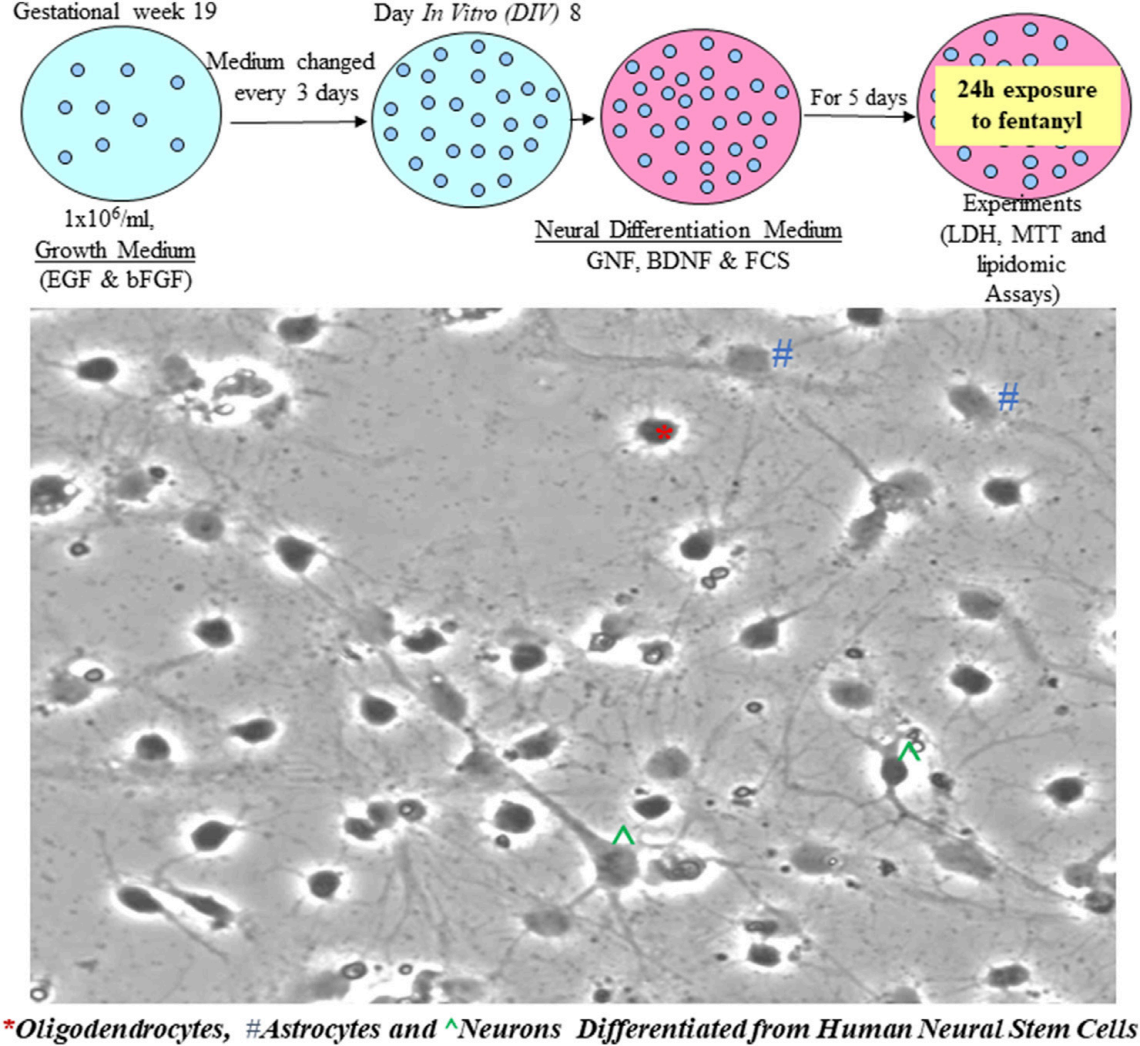
NSCs differentiated into neural cells when the cultures were maintained in neural differentiation medium. It is shown that most cells are differentiated with multiple processes and a neural network is formed. Based on their morphology, ^ represent a typical neuron with an axon and multiple dendrite processes; * represents a differentiated oligodendrocyte and # indicates an astrocyte.

Given the importance of NSC differentiation in the assessment of fentanyl-induced adverse effects/cell viability, starting from day *in vitro* 8 (DIV 8), NSCs were cultured in neural differentiation medium. After 5-day of differentiation, the differentiated neural/neuronal cells showed multiple processes, and a typical neural network was formed (Figure 2). Meanwhile, differentiated neurons, astrocytes, and oligodendrocytes (derived from human NSCs) could be morphologically identified (Figure 2).

### Cytotoxicity of fentanyl on NSCs and/or differentiated neural cells

**FIGURE 3:**
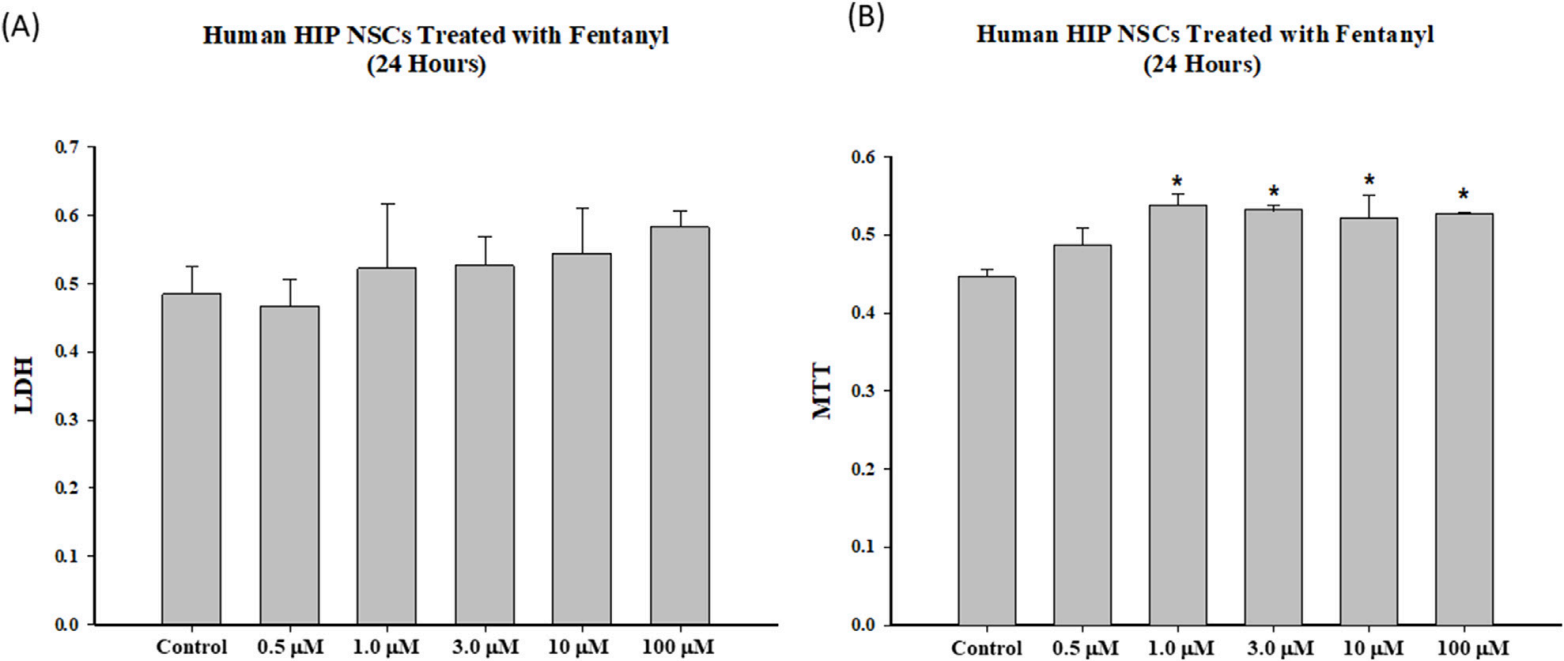
To determine the dose response effects and temporal relationships of fentanyl on cell viability, NSCs were exposed to fentanyl for 24 h at concentration of 0.5, 1.0, 3.0, 10 or 100 µM. Fentanyl exposure resulted in a slight dose-related increase (not significant) in the release of LDH **(A)** into the cell culture medium, compared with controls. No reduction in MTT **(B)** was observed in the fentanyl exposed group, compared with controls.

The LDH assays demonstrated that 24-h fentanyl exposure of NSCs at concentrations of 0.5, 1.0, 3.0, 10 or 100 µM resulted in a subtle, but a non-significant increase in the release of LDH into the cell culture medium. No significant MTT reduction was observed when the cultured NSCs were exposed to any of these five concentrations of fentanyl compared with controls (Figure 3). In contrast, 24-h fentanyl exposure at concentrations of 1.0, 3.0, 10 or 100 µM caused an elevation of MTT uptake (Figure 3).

**FIGURE 4:**
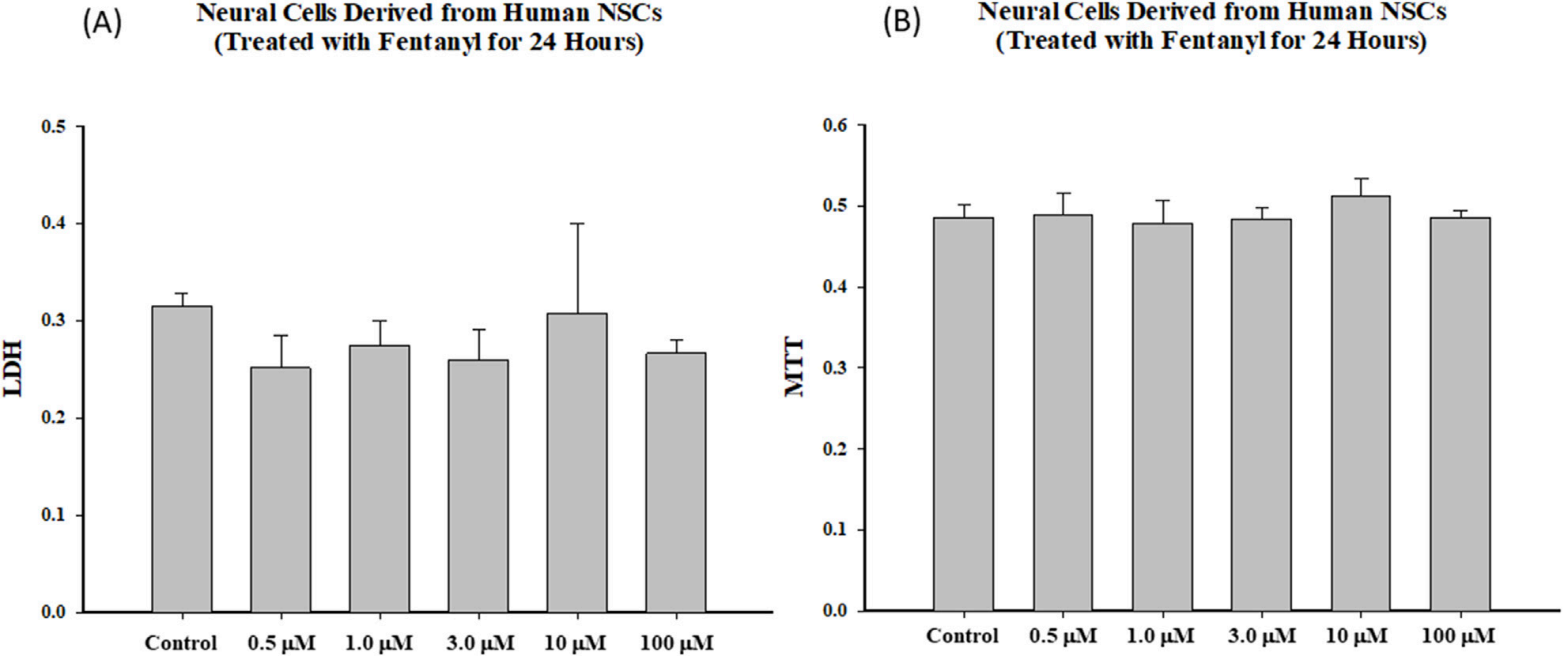
Exposure of differentiated cells to fentanyl for 24 h did not lead to changes in LDH release **(A)** or MTT reduction **(B)**. Each treatment condition was assessed at least in triplicate, and experiments were repeated three times independently.

Markers of cell death/damage were also used to assess the dose response effects of fentanyl exposure on the viability of differentiated neural cells. Like NSCs, fentanyl did not produce a remarkable increase in the release of LDH or a significant decrease of MTT uptake in the differentiated neural cells after 24-h exposure, compared with control (Figure 4). Each treatment condition was assayed at least in triplicate and the experiments were repeated three times independently.

### Lipidomic data

In total, 919 lipid species from 20 lipid classes were detected from differentiated cells treated with vehicle (Control, n = 6), or 1 µM (FL, n = 3), 10 µM (FM, n = 3) or 100 µM (FH, n = 3) fentanyl for 24 h (the experiments were repeated three times independently).

**FIGURE 5:**
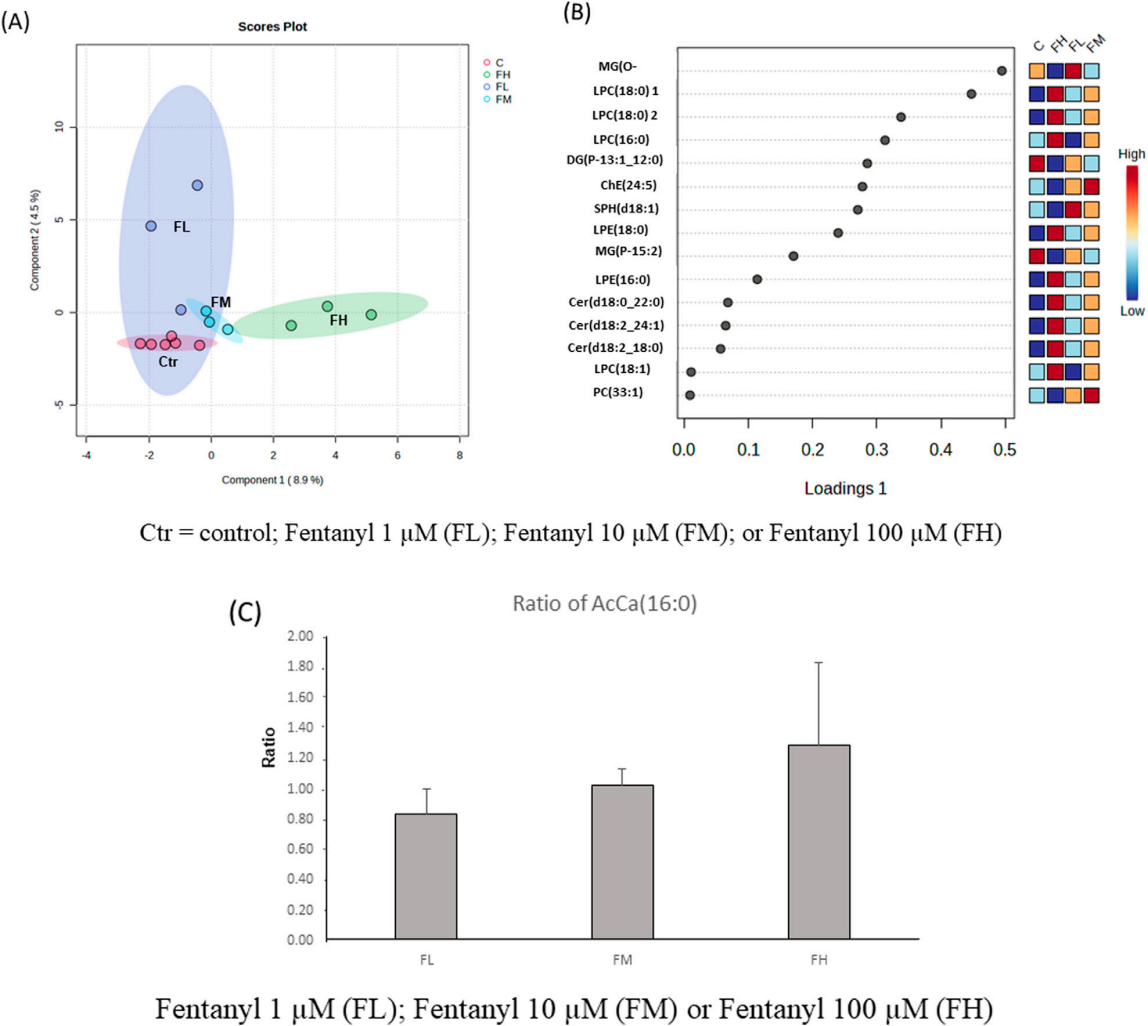
Principal component analysis (PCA) score plots **(A)**, the top 15 variable importance in projection (VIP) plot **(B)** of lipidomics data from differentiated neural cells (derived from NSCs) of control (Ctr), treated with fentanyl 1 µM (FL), fentanyl 10 µM (FM) or fentanyl 100 µM (FH), for 24 h. Dark red indicates that the normalized level is higher than the average; dark blue indicates that the normalize level is lower. C = control; Fentanyl 1 µM (FL); Fentanyl 10 µM (FM); or Fentanyl 100 µM (FH). Bar graphs of the ratio of AcCa (16:0) **(C)**, an intermediate metabolite of fatty acid *β*-oxidation in mitochondrial. Fentanyl 1 µM (FL); Fentanyl 10 µM (FM) or Fentanyl 100 µM (FH).

The Partial Least Squares Discriminant Analysis (PLSDA) effectively distinguishes between control and fentanyl-treated groups, and the high-dose group (FH) is located furthest from the control group (Figure 5A). The top 15 most variable important in projection (VIP) lipid species (based on VIP scores), which are responsible for the group separations, included LPC (16:0), LPC (16:1), LPC (18:1), CER (d18:0_22:0), CER (d18:2_18:0), CER(d18:2_24:1) which were significantly increased, while ChE (24:5) and SPH (d18:1) significantly decreased (Figure 5B). Despite these alterations, palmitoyl carnitine levels, indicative of mitochondrial function, remained unaffected by fentanyl exposure (Figure 5C), aligning with the findings from the MTT assays that showed no detectable mitochondrial damage after fentanyl exposure.

**FIGURE 6:**
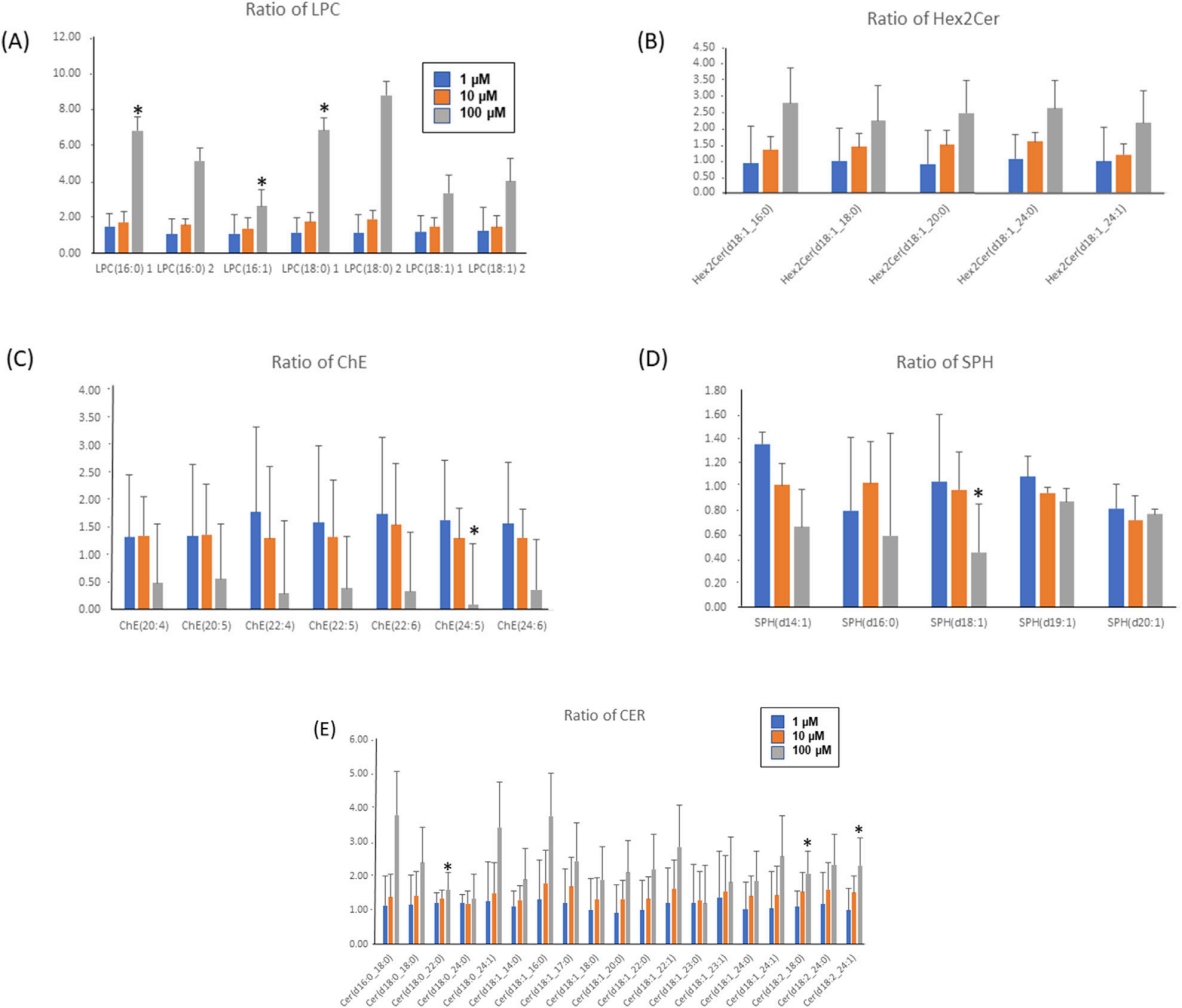
Bar graphs of the ratios of LPC **(A)**, Hex2Cer **(B)**, ChE **(C)**, SPH **(D)** and CER **(E)** lipids (treated/Ctr). Ratios of LPC, CER and Hex2Cer had a dose-dependent increase, while ratios of ChE and SPH lipids had dose-dependent decreases. Ratios of CER had a dose-dependent increase **(E)**. * Indicating ratios are significantly changed (vs. Control). Fentanyl 1 µM (FL, blue bar); Fentanyl 10 µM (FM, red bar) or Fentanyl 100 µM (FH, grey bar).

In Figure 6, the bar graphs delineate the dose-responsive modulation of lipid species upon fentanyl exposure, showcasing significant increments in LPCs (Figure 6A), CERs (Figure 6E), and di-hexosylceramides (Hex2Cer, Figure 6B), with the high-dose group (FH) demonstrating more than a twofold increase compared to the controls. Conversely, cholesterol esters (ChEs, Figure 6C) and sphingosines (SPHs, Figure 6D) manifest dose-dependent down-regulations, with reductions exceeding twofold in the high-dose scenario (Supplementary Table S1). This vividly illustrates the impacts of fentanyl on lipid metabolism, underscoring the substance’s capacity to induce profound alterations in cellular lipid profiles in a dose-dependent manner. It must be noted that only LPC (16:0), LPC (16:1), LPC (18:0), CER(d18:0_22:0), CER(d18:2_18:0), CER(d18:2_24:1) significantly increased, and only ChE(24:5) and SPH(d18:1) significantly decreased in the FH group vs. control.

**FIGURE 7:**
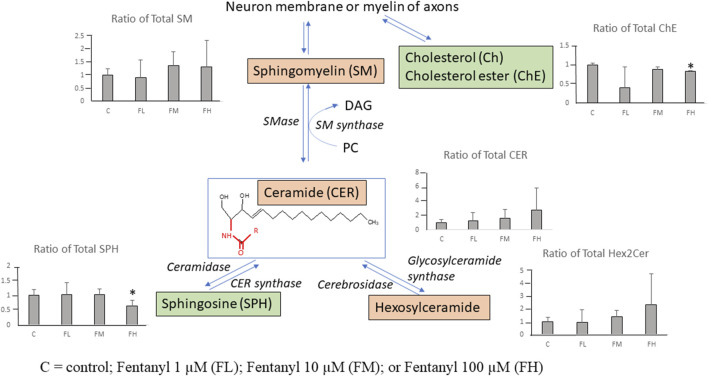
Summary illustration of the identified lipids involved in the ceramide pathways after fentanyl exposure, for 24 h. The scheme of the pathway is simplified and shows only relevant lipids. The bar graph shows the ratio of total lipid class level [(Sum (treated)/Sum (Control)]. The green (down-regulation) and pink (up-regulation) represent the changes in the total lipid class levels following fentanyl treatment. *Indicating ratios are significantly changed (vs. Control). C = control; Fentanyl 1 µM (FL); Fentanyl 10 µM (FM); or Fentanyl 100 µM (FH).

The affected lipids species involved in the CER pathways are summarized in Figure 7. Ratios of total lipid class of CER and Hex2Cer show dose-dependent increases.

Ratios of total lipid class of sphingomyelin (SM) had minor increases (∼1.3 fold) in the FH group vs. control. Ratios of total lipid class of ChE and SPH had significant decreases with <2-fold decreases in the FH group vs. control. Among the 20 detected lipid classes the total abundance of cholesterol ester and sphingosine classes significantly decreased while ceramide and hexosylceramide classes significantly increased (>2-fold increase) in the high-dose group (FH) vs. control. These lipidomic data indicate that the ceramide pathway might be disturbed by fentanyl treatment.

## Discussion

Accumulating data have suggested that an *in vitro* neuronal culture system could recapitulate some major events of CNS development *in vivo*. Culture models, such as NSC cultures, facilitate mechanistic dissections including potential fentanyl-induced neurotoxicity [14–16, 26–29, 30], which is difficult using *in vivo* systems. Originally, we hypothesized that: 1) application of *in vitro* NSC models and/or differentiated cells should be able to provide data that can inform clinical interventions and preclinical toxicology studies; 2) fentanyl-induced neural damage, if any, could depend on the dose given and the duration of exposure. In this study, commercially available and de-identified human NSCs were employed. The cultured cells exhibited typical features including self-renew and differentiation to generate neurons, astrocytes, and oligodendrocytes. Our data (Figures 1, 2) show that the model was successful, despite the minimal impact of fentanyl.

Fentanyl, a synthetic opioid, is widely used to supplement general anesthesia. The clinical response to given doses of an opioid varies considerably, and this is at least in part a result of variability in disposition [31]. Consequently, the influence of several factors, such as the patient’s age, sex, body weight, cardiac output, or type and duration of surgery and anesthesia, on the pharmacokinetics of opioids have been investigated [32–36]. In the present study, to determine dose-dependent effect curves and the temporal relationships between fentanyl exposures and NSC health, differentiation, and viability, a range of fentanyl at micromolar concentrations was tested. The selection of micromolar concentration was based on the fact that the reference concentrations in human blood (plasma) vary greatly depending on the doses, frequency, routes of administration, and as a primary anesthetic agent, for example, fentanyl is administered in very high doses during cardiac surgery [37–39]. Our data demonstrated that 24-h exposure of NSCs to fentanyl at micromolar concentrations did not significantly affect mitochondrial functions in NSCs, nor reduced cell viability. In fact, 24-h fentanyl exposure at concentrations of 1.0, 3.0, 10 or 100 µM even caused a certain level of elevation of MTT uptake into NSCs. Additionally, with up to 100 µM fentanyl no significant adverse effects were observed on cell viability (LDH) or mitochondrial health status (MTT). Therefore, these results suggest that at micromolar levels (concentrations) [32, 35], NSCs and immature neuronal cells were not sensitive to fentanyl-induced cell death, indicating the apoptotic cascades and acute neuronal damage (necrotic cascades/pathways) did not seem to contribute to the fentanyl-induced adverse effects.

Approximately 60% of the human brain is comprised of lipids. Despite this, the impact of chemicals on lipid composition is rarely considered. Lipidomics analysis was conducted to investigate whether the lipidome of neural cells derived from human NSCs was changed by fentanyl exposure. Currently, few studies have evaluated whether and/or how analgesics/anesthetics might affect the biodynamics of lipids [11, 36]. Identifying alterations of lipid composition may help to evaluate the adverse effects associated with fentanyl exposure at a variety of micromolar concentrations. Since neural/neuronal cells contain a wide variety of lipid classes and lipid species, lipidomic analysis using UHPLC/HRMS was conducted to investigate the impacts of different concentrations of fentanyl on neural/neuronal cells. Notably, the dose-dependent increases in LPCs and CERs, along with decreases in ChEs and SPH, hints at a disruption in the ceramide pathway, a potential mechanism for fentanyl-induced functional deficits and/or early states of neurotoxicity. Sphingolipids, the second most abundant membrane lipids after phospholipids [34], are classified into three lipid classes: ceramides, sphingomyelins (SM), and glycosphingolipids (Hex2Cer) [33] as displayed in Figure 7, where CER is a central point for both sphingolipid biosynthesis and catabolism. Sphingolipids play important roles in the development and maintenance of the functional integrity of the nervous system [40]. Dysregulated sphingolipid metabolism has been reported to be associated with a variety of neurodegenerative diseases [40–42], specifically accumulation of ceramides contributes to the neuroinflammatory process in a wide range of neurodegenerative diseases [40, 43, 44]. The sphingolipid pathway was disturbed as shown by dose-dependent increases in CER and Hex2Cer and decreases in SPH. These data indicate that the neural cells might experience inflammation associated changes caused by fentanyl exposure at 100 μM, in the absence of negative effects on LDH and MTT. The increases in CER and Hex2Cer and decreases in ChE by fentanyl might affect the structure, function, and stability of myelin, as well as for axonal growth of neurons. The lipidomic shifts underscore the intricate role of lipid metabolism in the neurotoxic effects associated with opioid exposure, particularly in pediatric settings. By highlighting the importance of the ceramide pathway and lipid metabolism, our findings advocate for a deeper exploration into how fentanyl alters cellular biochemistry, contributing to a more comprehensive understanding of their neurotoxic potential.

Given the paucity of research on how analgesics and anesthetics influence lipid biodynamics (crucial for brain structure and function), our lipidomics analysis using UHPLC/HRMS represents a significant step forward. Investigating the effect of various fentanyl dosages on neural cells derived from human NSCs has provided valuable insight into fentanyl’s effects at the cellular level, suggesting that lipidomic profiling provides complementary information to biological tests (for instances, LDH release and MTT assay) for evaluating the nuanced mechanisms of fentanyl-induced neurotoxicity and for developing strategies to protect vulnerable pediatric populations during opioid therapy. It should also be mentioned that the current study has some limitations. For example, 1) although each treatment condition was assayed at least in triplicate and the experiments were repeated three times independently, untargeted-lipidomics requires more experimental replicates than three replicates which matters in terms of statistical data analysis [45]. And 2) although, at our preliminary experiments, it is indicated that shorter periods of fentanyl exposure (including 1-, 3- and 6-h) did not induce dose-related adverse effects (data not shown) on NSC and differentiated neural cell viability, the effects of shorter exposure time on lipidomic profiles should be more impactful and should be addressed in our future fentanyl projects.

### Summary

MTT and LDH data showed that no detectable cell death occurred after fentanyl exposure at micromolar concentrations for 24 h. However, lipidomics analysis showed that the sphingolipid pathway was disturbed by fentanyl exposure at 100 µM for 24 h. Further, CER(d18:0_22:0), CER(d18:2_18:0) and CER (d18:2_24:1) may be potential biomarkers for neural cell inflammation status. Monitoring alteration of lipid composition and aberrant lipid metabolism may provide a more in depth understanding of the neurotoxic mechanisms, neuroinflammation, and neuronal viability/plasticity associated with fentanyl exposures.

## Data Availability

The original contributions presented in the study are included in the article/Supplementary Material, further inquiries can be directed to the corresponding author.

## Footnotes

1
www.metaboanalyst.ca
